# Outdoor Air Pollution, Preterm Birth, and Low Birth Weight: Analysis of the World Health Organization Global Survey on Maternal and Perinatal Health

**DOI:** 10.1289/ehp.1306837

**Published:** 2014-02-07

**Authors:** Nancy L. Fleischer, Mario Merialdi, Aaron van Donkelaar, Felipe Vadillo-Ortega, Randall V. Martin, Ana Pilar Betran, João Paulo Souza, Marie S. O´Neill

**Affiliations:** 1 University of South Carolina Arnold School of Public Health, Columbia, South Carolina, USA; 2 Department of Reproductive Health and Research, World Health Organization, Geneva, Switzerland; 3 Department of Physics and Atmospheric Science, Dalhousie University, Halifax, Nova Scotia, Canada; 4 School of Medicine, Universidad Nacional Autónoma de México, Mexico City, Mexico; 5 Instituto Nacional de Medicina Genomica, Mexico City, Mexico; 6 Harvard-Smithsonian Center for Astrophysics, Cambridge, Massachusetts, USA; 7 University of Michigan School of Public Health, Ann Arbor, Michigan, USA

## Abstract

Background: Inhaling fine particles (particulate matter with diameter ≤ 2.5 μm; PM_2.5_) can induce oxidative stress and inflammation, and may contribute to onset of preterm labor and other adverse perinatal outcomes.

Objectives: We examined whether outdoor PM_2.5_ was associated with adverse birth outcomes among 22 countries in the World Health Organization Global Survey on Maternal and Perinatal Health from 2004 through 2008.

Methods: Long-term average (2001–2006) estimates of outdoor PM_2.5_ were assigned to 50-km–radius circular buffers around each health clinic where births occurred. We used generalized estimating equations to determine associations between clinic-level PM_2.5_ levels and preterm birth and low birth weight at the individual level, adjusting for seasonality and potential confounders at individual, clinic, and country levels. Country-specific associations were also investigated.

Results: Across all countries, adjusting for seasonality, PM_2.5_ was not associated with preterm birth, but was associated with low birth weight [odds ratio (OR) = 1.22; 95% CI: 1.07, 1.39 for fourth quartile of PM_2.5_ (> 20.2 μg/m^3^) compared with the first quartile (< 6.3 μg/m^3^)]. In China, the country with the largest PM_2.5_ range, preterm birth and low birth weight both were associated with the highest quartile of PM_2.5_ only, which suggests a possible threshold effect (OR = 2.54; CI: 1.42, 4.55 and OR = 1.99; CI: 1.06, 3.72 for preterm birth and low birth weight, respectively, for PM_2.5_ ≥ 36.5 μg/m^3^ compared with PM_2.5_ < 12.5 μg/m^3^).

Conclusions: Outdoor PM_2.5_ concentrations were associated with low birth weight but not preterm birth. In rapidly developing countries, such as China, the highest levels of air pollution may be of concern for both outcomes.

Citation: Fleischer NL, Merialdi M, van Donkelaar A, Vadillo-Ortega F, Martin RV, Betran AP, Souza JP, O´Neill MS. 2014. Outdoor air pollution, preterm birth, and low birth weight: analysis of the World Health Organization Global Survey on Maternal and Perinatal Health. Environ Health Perspect 122:425–430; http://dx.doi.org/10.1289/ehp.1306837

## Introduction

Air pollution is associated with increased morbidity and mortality for multiple health indicators, including cardiovascular disease, lung cancer, acute respiratory infections, asthma, and pregnancy outcomes (Brunekreef and Holgate 2002; Glinianaia et al. 2004; Kampa and Castanas 2008; Lacasana et al. 2005; Maisonet et al. 2004; Šrám et al. 2005). Inequity in health outcomes associated with air pollution occurs among people living in low-income countries compared with high-income countries, and for poor people living in countries at all levels of development (O’Neill et al. 2008). Preterm birth (< 37 weeks gestation) and low birth weight (LBW) (< 2,500 g) have been associated with air pollution exposure, but the weight of the evidence is not yet sufficient to establish causality at this time (Maisonet et al. 2004; Šrám et al. 2005). LBW is a consequence of reduced length of gestation and/or restricted fetal growth *in utero* (Kramer 2003). Both prematurity and growth restriction make important contributions to morbidity and mortality during infancy, and in the long term these conditions may put adults at risk for a wide range of adverse health outcomes (Longo et al. 2013; Rogers and Velten 2011).

Air pollutants may be part of a complex set of factors that increase the risk of preterm birth or LBW through processes related to inflammation, oxidative stress, endocrine disruption, and impaired oxygen transport across the placenta (Slama et al. 2008). Exposure to airborne particles with diameter ≤ 2.5 μm (PM_2.5_) is of particular relevance in relation to pregnancy outcomes. These particles can be inhaled into the deep regions of the lung, and oxidative stress and inflammation may be among the mechanistic pathways through which exposure to this pollutant may contribute to onset of preterm labor (Slama et al. 2008). In addition, previous research shows that fine particles are more spatially homogeneous than other pollutants, and outdoor measurements of these particles may serve as a useful proxy index of personal exposure to a range of pollutants (Sarnat et al. 2005).

Most studies of air pollution and adverse birth outcomes have been conducted in communities in high-income countries, with very few data in low- and middle-income countries. Few studies have examined cross-country comparisons of the relationship between air pollution and birth outcomes, where differences in pollution levels may be most extreme. The World Health Organization (WHO) Global Survey on Maternal and Perinatal Health (WHOGS) database (Shah et al. 2008) offers a unique opportunity to link global estimates of fine particulate matter with pregnancy outcomes in many areas of the world where this line of investigation has yet to be undertaken.

The aim of this paper is to examine the relationship between PM_2.5_ and preterm birth and LBW among 22 countries in the WHOGS.

## Methods

*Population*. The WHOGS is a multicountry, cross-sectional survey that collected data on all deliveries in participating facilities for 2–3 months, depending on the annual volume of deliveries of the facility. Data were collected for > 290,000 women in 373 institutions in 24 countries in Africa, Latin America, and Asia. The WHOGS was implemented in Africa and the Americas between September 2004 and March 2005, and in Asia between October 2007 and April 2008. The survey had a stratified multistage cluster sampling design, with four countries sampled from each of the 14 WHO-defined subregions that are under the broader regions of Africa, the Americas, and Asia (except in two subregions with only three countries each). The capital city and two randomly selected provinces were included, followed by a random sampling of up to seven health institutions in each location with at least 1,000 deliveries in the year before the survey. Facility data on available services were collected at each site, as were data on all women who delivered in the facilities during the study period. Individual-level data were abstracted from medical records by trained data collectors. We obtained written permission from the ministry of health of each country and the director of each health facility. Individual informed consent was not obtained because this study was a cluster-level study in which data were extracted from medical records with no individual identification. The ethics review committee of WHO and that of each country approved the study protocol. Detailed methodology of the WHOGS has been described elsewhere (Shah et al. 2008).

The WHOGS defined preterm birth as gestational age of < 37 weeks at delivery, as determined by the best available obstetric estimate of gestational age. LBW was defined as < 2,500 g at birth. Because of heterogeneity in the quality of the estimated gestational age across the survey countries, we did not use the traditional cut-off of LBW being among only full-term births. Only live, spontaneous, singleton births were included in the analyses. All analyses were restricted to facilities with < 20% prevalence of preterm birth (to maintain as much comparability as possible for the estimation of gestational age) and > 100 births recorded during the 2- to 3-month sampling period (having < 100 suggests problems with the completeness of sampling of births or that the facility may have had fewer than the inclusion criterion of 1,000 deliveries/year). In addition, countries with fewer than half of the randomly selected facilities from that country meeting our inclusion criteria were also excluded. Data from five African countries, eight countries in the Americas, and nine Asian countries are included in this analysis (22 of 24 countries in the WHOGS).

Demographic and pregnancy-related factors, including age, maternal education (years), parity, prenatal care (number of antenatal visits), and infant sex were treated as potential confounders in the analysis. All variables were continuous in the models, except infant sex. Women with missing data on the birth outcomes or any of the potential confounders were excluded from the analysis. A median of 0.7% of women per facility were missing data for preterm birth, with 0.4% per facility missing for LBW. Data on the birth outcomes were rarely missing; education was the most common source of missing data, although information on prenatal care was also frequently missing in some countries. Of the 305 facilities in the analysis, 43 had > 10% of women without complete data on the birth outcomes and potential confounders. Sensitivity analyses were run excluding the 43 facilities, in addition to two other facilities from one country since all of its other facilities had high levels of missingness.

*Air pollution exposure assessment and other indicators*. Remote sensing data provide a useful estimate of pollution levels in the absence of extensive local ground-based monitor networks, particularly when the nearest monitor is located > 100 km away (Lee et al. 2012). Such monitoring networks are rare in less wealthy regions of the world (Cohen et al. 2005). Air pollution exposure for this study is therefore represented with global estimates of PM_2.5_ as developed by van Donkelaar et al. (2010). These values provide a long-term average (2001–2006) global estimate of PM_2.5_ at approximately 10 km × 10 km resolution. They are derived from a combination of observations from the Moderate Resolution Imaging Spectroradiometer (MODIS) (Levy et al. 2007) and Multiangle Imaging Spectroradiometer (MISR) (Diner et al. 1998) instruments from the Terra satellite, and simulations with the GEOS-Chem chemical transport model (www.geos-chem.org). The resultant PM_2.5_ data were validated using ground-based data and have an expected 1-sigma uncertainty of 1 μg/m^3^ + 25%. Further details have been published elsewhere (van Donkelaar et al. 2010).

Our objective was to use these PM_2.5_ concentrations to estimate exposure during pregnancy among the women whose data were captured by the WHOGS. Health facilities participating in the WHOGS were geocoded using the exact address or city information, as available, to determine the closest possible geographical coordinates using Google Earth (http://www.google.com/earth/). Next, 50-km–radius circular buffers were created around the coordinates of interest, and average PM_2.5_ concentrations within these buffers were then matched to the health facilities. This buffer size was chosen to represent a realistic distance within which women giving birth at the facilities might live, because residential addresses of these women were not available in the survey. The seasonal impact of sampling and uncertainty on satellite-derived PM_2.5_ estimates unfortunately limited their direct use on a monthly basis. Rather, adjustment was made for the impact of seasonality on the relationship between PM_2.5_ levels and adverse birth outcomes using simulated seasonality from the GEOS-Chem model. A scalar variable of PM_2.5_ deviation from the overall 2001–2006 average was simulated for each calendar month and multiplied by the original PM_2.5_ level to estimate exposure for the calendar month preceding each woman’s delivery date. The seasonally adjusted PM_2.5_ values were used in the regression analyses. The month before birth was chosen for seasonal adjustment because of the strong seasonal patterns of air pollution exposure in some locations, and the potential importance of exposure during the third trimester to adverse birth outcomes (Ritz and Wilhelm 2008; Woodruff et al. 2009). Exposure in the first trimester has also been associated with adverse birth outcomes. Therefore, we also performed sensitivity analyses using an average of the scalar variable from the first 3 months of pregnancy to adjust for seasonal variation from the overall average.

For some locations, data were available from air pollution monitors located within 50 km of the clinics. In these cases, a comparison between PM_2.5_ levels measured by the ground monitors and the levels estimated from the satellite imagery was possible using supplemental data published with the original satellite estimates publication (van Donkelaar et al. 2010). We calculated ratios of the measured to estimated PM_2.5_ and averaged the ratios for the corresponding metropolitan area. In the instance where the ratio was either > 2.0 or < 0.50, we added half the difference between the average of the measured concentrations in the metropolitan area near the clinic and those estimated from the remote sensing imagery, and incorporated this adjusted estimate in sensitivity analyses. After calculating the ratio of available ground-based monitored PM_2.5_ and satellite-estimated data, only one study city, São Paulo, Brazil, had at least a 2-fold difference between the methods for the second sensitivity analysis. Its ratio of measured concentration to satellite estimates was 2.6. Thus, for the Brazilian clinics near São Paulo, 8.24 μg/m^3^ was added to the satellite-derived estimate of PM_2.5_ within 50 km of the clinic. Sensitivity analyses were run using the adjusted Brazil estimates.

We also examined several country-level indicators in relation to adverse birth outcomes and air pollution levels. Gross domestic product (GDP) per capita [in international dollars at purchasing power parity (PPP) rates] was obtained from the Central Intelligence Agency’s World Factbook (Central Intelligence Agency 2007), and population living in urban areas (percent), per capita health care expenditure (current U.S. dollars), and the country-level Gini coefficient, a measure of income inequality with values from 0 (equality) to 1 (inequality), were obtained from the World Bank’s World Development Indicators (World Bank 2009), except for the Gini coefficient data for Algeria, Cuba, and Japan, which were obtained from the World Income Inequality Database (United Nations University–World Institute for Development Economics Research 2008). Data from 2006 were used for all country-level variables, or the closest year if data were unavailable for 2006.

*Statistical analysis*. Birth outcomes for women from the same health facilities may be correlated, thereby violating the independence assumption of basic regression models. Therefore, we used generalized estimating equation (GEE) models (Liang and Zeger 1986) to account for the nested structure of the data (individual women within health facilities within countries) when estimating the associations between seasonally adjusted, clinic-level PM_2.5_ exposure levels and birth outcomes. Two GEE models were run for each outcome. In the first model, a global estimate was obtained combining all countries while controlling for mother’s age, education, parity, and prenatal care and the infant’s sex. The second model was also adjusted for other country-level covariates (GDP per capita, urbanicity, antenatal care coverage, per capita health care expenditure, and the Gini coefficient) when determining the global estimate. PM_2.5_ effect estimates were calculated per 10-μg/m^3^ increments and as quartiles, in separate models. The quartiles were based on the distribution for the entire study population.

We ran corresponding country-specific GEE models for China and India, the two countries with the widest ranges of PM_2.5_ levels. New quartile cut points for these models were based on country-specific distributions.

## Results

**Figure 1:**
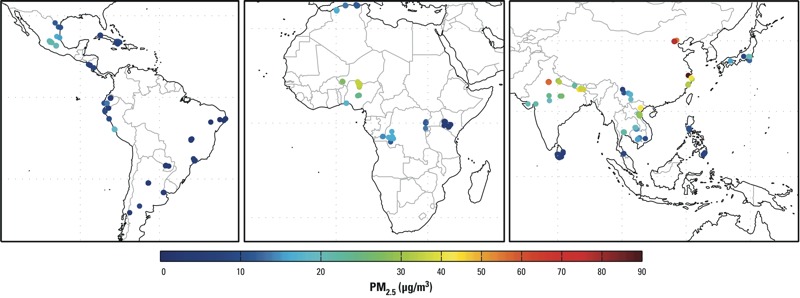
Map showing estimated PM_2.5_ levels in 50-km–radius buffers around clinics in 22 countries, 2001–2006.

Data from 192,900 live, spontaneous, singleton births from 22 countries in Africa, Asia, and Latin America were used in our analyses (Table 1). The prevalence of preterm birth ranged from 3.0% in Vietnam, to 11.1% in Thailand. Algeria had the lowest prevalence of LBW at 3.5%, and India had the highest at 20.4%. Paraguay had the lowest facility-level average PM_2.5_ levels during 2001–2006. Facilities in China and India had the largest ranges of average PM_2.5_, and also the facilities with the highest average levels. PM_2.5_ levels averaged across 2001–2006 for each facility can be seen in Figure 1.

**Table 1:** Description of preterm birth and air pollution characteristics, by country, WHO Global Survey on Maternal and Perinatal Health, 2004–2008.

Region and country	No. of facilities	Live, spontaneous, singleton births (*n*)	Preterm birth (%)	LBW (%)	Mother’s age [years (mean ± SD)]	Mother’s education [years (mean ± SD)]	Parity (mean ± SD)	Antenatal visits (mean ± SD)	Mean PM_2.5_ [μg/m^3^ (range)^*a*^]	Seasonally adjusted PM_2.5_ [μg/m^3^ (range)]
Africa
Algeria	17	12,718	3.7	3.5	30.3 ± 5.8	8.6 ± 4.6	2.6 ± 1.7	5.0 ± 2.6	10.7–16.7	3.6–11.7
Congo, DR	19	7,067	7.4	11.8	27.2 ± 6.8	8.3 ± 3.8	3.3 ± 2.4	3.6 ± 1.5	11.7–16.8	6.6–24.4
Kenya	19	16,694	9.4	7.3	24.7 ± 5.7	9.8 ± 2.9	2.1 ± 1.4	4.1 ± 2.3	4.2–5.5	2.5–7.7
Niger	7	4,826	3.2	10.4	26.6 ± 6.5	3.8 ± 4.4	3.4 ± 2.4	2.9 ± 1.5	27.7–34.1	3.0–44.0
Nigeria	17	6,538	8.6	5.3	27.6 ± 6.0	9.4 ± 5.6	3.0 ± 2.2	5.7 ± 3.9	17.5–35.4	7.1–53.5
Asia
Cambodia	5	5,170	5.6	6.9	26.8 ± 5.5	7.0 ± 3.8	1.8 ± 1.2	4.4 ± 2.1	13.3–15.8	16.8–23.9
China	21	9,221	4.8	3.6	26.2 ± 4.7	8.7 ± 3.5	1.4 ± 0.6	5.9 ± 3.5	6.4–98.1	2.6–145.2
India	13	14,622	10.3	20.4	24.5 ± 3.6	6.1 ± 4.1	1.8 ± 1.0	3.2 ± 2.8	19.6–63.9	10.6–109.3
Japan	10	2,191	4.3	7.9	31.0 ± 4.9	14.0 ± 2.0	1.6 ± 0.8	11.8 ± 3.2	8.7–20.9	11.8–34.9
Nepal	8	7,042	9.2	11.4	23.5 ± 4.2	6.1 ± 4.6	1.6 ± 0.9	3.8 ± 2.1	20.6–46.3	11.7–61.6
Philippines	17	11,326	7.6	14.1	26.3 ± 6.4	10.4 ± 2.6	2.2 ± 1.6	4.5 ± 2.8	8.2–11.0	12.7–23.2
Sri Lanka	14	6,381	8.1	14.2	27.6 ± 5.5	10.4 ± 2.6	1.9 ± 1.0	8.6 ± 3.3	5.5–7.7	4.9–14.9
Thailand	11	7,344	11.1	9.2	26.9 ± 6.1	9.6 ± 4.0	1.7 ± 0.9	7.9 ± 3.6	8.9–21.7	15.8–35.6
Vietnam	15	11,800	3.0	4.1	27.7 ± 4.7	12.4 ± 2.8	1.6 ± 0.7	5.8 ± 2.8	9.6–43.4	12.1–54.3
Latin America
Argentina	14	7,745	6.6	5.7	26.6 ± 6.5	9.3 ± 3.1	2.3 ± 1.7	6.2 ± 3.0	4.5–8.3	3.6–10.1
Brazil	19	10,735	7.1	8.4	24.1 ± 6.0	7.6 ± 3.1	2.2 ± 1.6	5.9 ± 2.6	1.4–6.3	1.3–9.3
Cuba	17	7,841	4.0	3.9	26.3 ± 6.4	11.4 ± 2.6	1.7 ± 0.8	11.2 ± 2.7	7.1–9.2	3.7–8.5
Ecuador	13	9,845	7.0	10.3	24.6 ± 6.3	9.0 ± 3.6	2.3 ± 1.5	5.3 ± 3.0	4.0–13.0	2.8–14.1
Mexico	20	14,497	7.5	7.5	25.0 ± 6.0	8.4 ± 3.3	2.2 ± 1.4	6.5 ± 3.0	10.8–21.9	4.3–20.1
Nicaragua	6	4,001	6.3	7.0	23.0 ± 5.8	6.8 ± 3.6	2.2 ± 1.5	4.0 ± 2.6	7.2–8.1	1.0–10.9
Paraguay	6	2,466	8.6	5.6	25.4 ± 6.4	8.8 ± 3.4	2.4 ± 1.8	4.9 ± 2.9	3.3–4.5	3.1–6.8
Peru	17	12,830	5.8	5.0	26.2 ± 6.4	10.1 ± 3.1	2.0 ± 1.3	6.2 ± 3.1	8.6–20.6	6.9–30.9
DR, Democratic Republic. ^***a***^Mean PM_2.5_ refers to the mean values during 2001–2006 for each facility in each country.										

The odds of preterm birth among women exposed to higher seasonally adjusted PM_2.5_ levels were not different from those for women exposed to lower levels of PM_2.5_ based on models with and without adjustment for country-level variables (Table 2). When assessing the results by PM_2.5_ quartiles, odds ratios (ORs) were close to the null for all exposure quartiles, without evidence of a positive trend.

**Table 2:** Adjusted ORs (95% CI) for preterm birth and LBW associated with a 10-μg/m^3^ increase in PM_2.5_ and with quartiles of PM_2.5_ (relative to the lowest quartile) after adjusting exposure estimates to account for seasonality, WHO Global Survey on Maternal and Perinatal Health, 2004–2008.

Outcome	Model 1	Model 2
Preterm birth
PM_2.5_ (10 μg/m^3^)	0.96 (0.91, 1.02)	0. 96 (0.90, 1.02)
< 6.35	1.0 (Reference)	1.0 (Reference)
6.35 to < 12.32	1.08 (0.95, 1.22)	1.08 (0.95, 1.24)
12.32 to < 22.20	1.05 (0.90, 1.23)	1.06 (0.90, 1.25)
≥ 22.20	0.96 (0.79, 1.17)	0.96 (0.79, 1.18)
LBW
PM_2.5_ (10 μg/m^3^)	1.00 (0.97, 1.03)	0.99 (0.96, 1.01)
< 6.298	1.0 (Reference)	1.0 (Reference)
6.298 to <11.96	1.06 (0.97, 1.16)	1.05 (0.95, 1.16)
11.96 to <20.16	1.19 (1.06, 1.33)	1.15 (1.02, 1.30)
≥ 20.16	1.22 (1.07, 1.39)	1.15 (1.01, 1.32)
All models are GEE models with a logit link. All models were adjusted for mother’s age, education, parity, prenatal care, and infant’s sex. Model 2 also adjusted for country-level variables GDP per capita, urbanicity, health care expenditure per capita, and Gini coefficient. Models for 10 μg/m^3^ PM_2.5_ and quartiles of PM_2.5_ were run separately. PM_2.5_ levels were seasonally adjusted.		

For LBW, women in the highest two quartiles had higher odds of LBW babies compared with women in the lowest quartile of PM_2.5_ exposure [OR = 1.19; 95% CI: 1.06, 1.33 for quartile 3 (PM_2.5_ 11.96 to < 20.16 μg/m^3^) vs. quartile 1 (PM_2.5_ < 6.298 μg/m^3^); OR = 1.22; 95% CI: 1.07, 1.39 for quartile 4 (PM_2.5_ ≥ 20.16 μg/m^3^) vs. quartile 1 (PM_2.5_ < 6.298 μg/m^3^)] (Table 2). These results were slightly attenuated, but remained statistically significant, when adjusted for country-level variables.

Because of the large variability of PM_2.5_ levels in China and India, we examined each country separately using country-specific quartiles of exposure (Table 3). In China, we found a higher odds of preterm birth and LBW among mothers in the highest quartile of PM_2.5_ exposure (≥ 36.5 μg/m^3^) compared with those in the lowest quartile (< 12.5 μg/m^3^) (OR = 2.54; 95% CI: 1.42, 4.55 for preterm birth; OR = 1.99; 95% CI: 1.06, 3.72 for LBW). Linear trends based on PM_2.5_ modeled as a simple continuous variable were also statistically significant for each birth outcome in China (OR = 1.11; 95% CI: 1.04, 1.17 and OR = 1.07; 95% CI: 1.01, 1.14 for preterm and LBW in association with 10-μg/m^3^ increases in PM_2.5_, respectively). In India, we saw evidence for an inverse association between PM_2.5_ levels and both preterm birth and LBW. Results for preterm birth were not statistically significant for either the linear estimate or the quartile analysis. However, for the quartile analysis of LBW we saw an inverse association, whereby women in the highest quartile of PM_2.5_ (≥ 70.3 μg/m^3^) exposure had a lower odds of LBW babies compared to women in the lowest quartile (< 18.8 μg/m^3^) (OR = 0.82; 95% CI: 0.75, 0.90). The linear trend for this relationship was also statistically significant.

**Table 3:** Adjusted ORs (95% CI) for preterm birth and LBW associated with 10-μg/m^3^ PM_2.5_ and with quartiles of PM_2.5_ (relative to the lowest quartile) after adjusting exposure estimates to account for seasonality for China and India, WHO Global Survey on Maternal and Perinatal Health, 2004–2008.

Exposure	Preterm birth	LBW
China
PM_2.5_ (10 μg/m^3^)	1.11 (1.04, 1.17)	1.07 (1.01, 1.14)
< 12.5	1.0 (Reference)	1.0 (Reference)
12.5 to < 17.7	0.77 (0.45, 1.13)	0.98 (0.70, 1.39)
17.7 to < 36.5	0.97 (0.70, 1.34)	1.08 (0.84, 1.40)
≥ 36.5	2.54 (1.42, 4.55)	1.99 (1.06, 3.72)
India
PM_2.5_ (10 μg/m^3^)	0.96 (0.91, 1.03)	0.97 (0.95, 0.99)
< 18.8	1.0 (Reference)	1.0 (Reference)
18.8 to < 35.3	1.08 (0.87, 1.34)	1.01 (0.96, 1.07)
35.3 to < 70.3	0.92 (0.72, 1.19)	0.90 (0.79, 1.02)
≥ 70.3	0.76 (0.49, 1.17)	0.82 (0.75, 0.90)
All models are GEE models with a logit link. All models were adjusted for mother’s age, education, parity, prenatal care, and infant’s sex. Models for 10 μg/m^3^ PM_2.5_ and ­quartiles of PM_2.5_ were run separately. PM_2.5_ levels were seasonally adjusted.		

We ran sensitivity analyses excluding facilities with a high level of missingness and for PM_2.5_ adjusted from ground-based monitors. For preterm birth, results were comparable to the main analysis when we excluded facilities with the large proportions of births with missing data, and when we adjusted exposure levels for women who gave birth in Brazilian clinics near São Paulo using data from ground-based monitors; there was no evidence that preterm birth was associated with PM_2.5_ (data not shown). For LBW, results from the sensitivity analyses were qualitatively similar to the main analysis, though model 2 ORs were no longer statistically significant (α = 0.05) for the upper two quartiles compared with the lowest quartile (data not shown).

When we performed sensitivity analyses of associations with PM_2.5_ levels that were adjusted for levels during the first trimester rather than levels during the month before birth, associations of preterm birth and LBW with PM_2.5_ modeled as a continuous variable were negative for model 2 (OR = 0.59; 95% CI: 0.49, 0.70 and OR = 0.75; 95% CI: 0.68, 0.83 for a 10-μg/m^3^ increase in exposure for preterm birth and LBW, respectively). For both outcomes, ORs for exposures in the second and third quartiles versus the first quartile (< 4.8 μg/m^3^) were nonsignificant in all models, but the highest quartile of PM_2.5_ exposure (≥ 27.3 μg/m^3^) was negatively associated with preterm birth and LBW (OR = 0.25; 95% CI: 0.09, 0.67 and OR = 0.59; 95% CI: 0.38, 0.92, respectively, for model 2.)

## Discussion

We investigated the relationship between air pollution and pregnancy outcomes across countries from vastly different regions of the world. By using data from women in the WHOGS and PM_2.5_ levels derived from remote sensing data, we were able to estimate associations for a study population that included women from areas of the world where it is often difficult to acquire reliable data on both pregnancy outcomes and air pollution concentrations. Estimated PM_2.5_ exposures were not associated with preterm birth based on our analysis, but LBW was significantly higher among women who delivered in facilities where PM_2.5_ concentrations were above the median (i.e., > 12.0 μg/m^3^) compared with women delivering at facilities with average PM_2.5_ levels < 6.3 μg/m^3^. In China, the country with the largest range of PM_2.5_ exposure levels, both preterm birth and LBW were significantly higher among women with estimated exposure to at least 36.5 μg/m^3^ of PM_2.5_ compared with women in the lowest quartile of exposure (< 12.5 μg/m^3^).

For preterm birth, we found null results when looking at PM_2.5_ levels across countries. In the United States and other high-income countries, PM_2.5_ has been associated with preterm birth in many studies (Brauer et al. 2008; Chang et al. 2012; Darrow et al. 2009; Huynh et al. 2006; Kloog et al. 2012; Ritz et al. 2007; Wu et al. 2009, 2011), although two studies reported no association (Gehring et al. 2011; Rudra et al. 2011). Few studies have been published on the relation between PM_2.5_ and preterm birth in low- and middle-income countries, or across countries at different levels of development. PM_10_ was associated with preterm birth in a study of > 374,167 births from Seoul, South Korea, in 1998–2000 using Cox models and exposure by trimester (Suh et al. 2009). In China, PM_10_ was associated with preterm birth in a time series analysis of daily births in 2004 in Shanghai (*n* = 3,346 preterm births) (Jiang et al. 2007), and in a time series analysis of 142,312 births in 2007 in Guangzhou (Zhao et al. 2011). Misclassification of the exposure or preterm birth or uncontrolled confounding by co-exposures in this sample of mostly low- and middle-income countries could have biased associations toward the null in our analysis. In China, only the highest quartile of PM_2.5_ exposure was associated with preterm birth compared with the lowest quartile. It may be that, given co-exposures to other environmental factors—which may act as uncontrolled confounders (e.g., poor nutrition due to seasonal availability) or effect modifiers (e.g., indoor air pollution) of the relationship—the impact of air pollution may be most prominent at higher levels in middle-income countries, of which China is an important example. The null results in India could be attributable to a downward bias due to misclassification of the pollution exposure or the measurement of preterm birth, or due to other co-exposures or environmental factors, as described above. Another possibility is that the most severely affected fetuses did not survive to be counted as live births, resulting in the appearance of protective effects in the highest exposure categories. In addition, the quartile cut points in both China and India were much higher than the cut points from the overall analysis. Exposure in the lowest quartile in China and India may have been so high already that a relationship between PM_2.5_ exposure and preterm birth and LBW would not be detectable with the first quartile as the reference level of exposure.

LBW was positively associated with PM_2.5_ exposure when data were pooled across all 22 countries in our analysis, consistent with findings in the United States and other high-income countries showing an increased risk of LBW with higher levels of PM_2.5_ (Bell et al. 2007; Kloog et al. 2012; Morello-Frosch et al. 2010; Parker et al. 2005; Wilhelm et al. 2012); this increase was also evident in a recent meta-analysis of data from nine mostly high-income countries (Dadvand et al. 2013). Other studies, however, have found no relationship (Brauer et al. 2008; Gehring et al. 2011). Again, evidence from low- and middle-income countries is scarce. A study of 891 newborns born 1994–1999 and randomly selected from among participants in a case–control study from two districts in the Czech Republic found an increased risk of LBW associated with PM_2.5_; analyses did not adjust for potential confounders (Rossner et al. 2011). Other studies looking at PM_10_ found a higher risk of LBW associated with exposure. A cross-sectional study in São Paulo, Brazil, of 179,460 live births during 1997 found that PM_10_ exposure during the first trimester of pregnancy was associated with LBW (Gouveia et al. 2004). However, a cross-sectional study of births from 2002 (*n* = 77,987) in Rio de Janeiro, Brazil, reported no association between PM_10_ (PM with diameter ≤ 10 μm) exposure and LBW, regardless of trimester of exposure (Junger and de Leon 2007). In Seoul, South Korea, a cross-sectional study of births from 2002–2003 found a higher risk of LBW associated with annual PM_10_ exposure (Seo et al. 2007); a similar study of 177,660 births from 2004 in seven Korean cities found the same relationship (Seo et al. 2010). We again saw evidence for a threshold effect for LBW in China, where women exposed to at least 36.5 μg/m^3^ of PM_2.5_ had higher odds of experiencing LBW. In India, contrary to expectation, women in the highest quartile of PM_2.5_ exposure experienced a lower risk of LBW compared to women in the lowest quartile. This may be attributable to a number of factors, including co-exposures (such as indoor air pollution) or residual confounding (such as malnutrition). These factors may outweigh any potential effect of outdoor air pollution that we would expect to see. Also, as noted above, other potential explanations include selective survival of fetuses that were not as severely affected, and also that the quartiles cut points are higher in China and India than in the overall analysis, resulting in comparisons with lowest quartiles that themselves contained fairly high exposure levels.

*Limitations and strengths*. This study has a number of limitations. The survey data were cross-sectional, so we were unable to assess the dynamic relationship between variations in preterm birth and other adverse outcomes, and their relationships with air pollution over time. Related to this, the exposure assessment is a 6-year average of particulate matter rather than a point-specific exposure assessment. We did have some data mismatch in timing, since the exposure was assessed for 2001–2006, whereas some of the birth data were collected from 2007 through 2008 (Asia). We treated the 6-year average exposure as a proxy for long-term exposure. Because pollution levels are typically correlated over time, the use of the 6-year average data as a proxy should be representative of the period during which births in Asia were recorded. If pollution levels increased significantly in the Asian countries in 2007–2008, the association between pollution and adverse birth outcomes would be underestimated. A further limitation of the data mismatch was that the WHOGS was collected only during particular months in each region. Since pregnancy outcomes and air pollution show seasonal variation, we may not have captured significant changes in weather that may have occurred annually—for example, that would alter the relationship between air pollution and adverse birth outcomes.

Additionally, there may be critical periods during the pregnancy when fetuses are particularly vulnerable to the effects of air pollution. We did adjust our pollution estimates for seasonal differences, to help account for some of these issues. However, when we conducted a sensitivity analysis using the first trimester as the critical period for seasonal adjustment rather than the month before birth, we found null and protective effects, contrary to expectation. Because the first trimester was determined by subtracting the gestational age from the birth date, the period may not have been accurately obtained given the potential issues with determination of gestational age (see more details below). In addition, because the sampling of births was done during specific months in each region, and generally excluded the months April–May through August–September, we do not have a full picture of the exposures throughout the year. If annual fluctuations were important during the years of sampling, we may not be accurately capturing the true exposure.

We also assumed that the particulate matter measurement at the facility is representative of the exposure to each of the women who delivered at the facility. By including a buffer of 50 km around the clinic, we tried to ensure that most women who used the clinic had an appropriate exposure value assigned to them. Because PM_2.5_ is one of the most spatially homogeneous markers of air pollution, this assumption is often applied in air pollution epidemiology (Miller et al. 2007; Park et al. 2006). Additionally, these particles can be well correlated with individual exposures (Sarnat et al. 2005). However, it is possible that women traveled > 50 km to the clinics. For this reason and others, exposure could have been misclassified.

Misclassification is also possible for the outcome variables, preterm birth and LBW. The capacity to accurately measure some variables in resource-poor countries (e.g., gestational age and birth weight) is a well-understood challenge. Although gestational age was calculated by the best available obstetric estimate at each clinic, the precision of this estimate may vary between clinics within countries, and between countries. Because the definition of preterm birth relies on gestational age, it may have been misclassified. We attempted to minimize the misclassification of LBW by not restricting the definition to full term infants, although misclassification may still exist.

We also have limited data on individual characteristics. Smoking information was not collected in the survey, and the only measure of the mother’s weight was “latest weight before delivery,” the date of which was not recorded; so we were unable to accurately calculate body mass index for the women and thus did not include it as a confounder. We also had no information on indoor air pollutants, which would be particularly important in the poorer countries where women often cook with biomass fuels indoors. Because most of the countries in our study are low- and middle-income countries, this is of particular concern. Other limitations to conducting these types of studies in poorer countries, and across countries at different levels of development, are that there may be other area-level confounders that affect the relationship of interest. For instance, season may affect both air pollution levels and nutrient availability [e.g., antioxidant vitamins (Casanueva et al. 2005)], which certainly affect pregnancy outcomes. Poorer countries are also more like to have worse air quality (with fewer regulations restricting pollution) and more vulnerable populations in general (Cohen et al. 2005). We tried to account for some of these between-country variations by including country-level markers of economic development and inequality, but we recognize that these may not be sufficient controls for these differences.

Despite the limitations, this study has many strengths. This is the first multicountry study to analyze air pollution as a potential determinant for preterm birth and LBW that included data from predominantly low- and middle-income countries (22 countries in three different regions). An additional strength is the homogeneity of the design and data collection across countries through a standardized form and training for data collection.

## Conclusions

This study is the first to investigate the relationship between air pollution and adverse pregnancy outcomes using WHOGS data from mostly low- and middle-income countries from around the world. We found no association between PM_2.5_ levels and preterm birth, but higher PM_2.5_ levels were associated with a higher risk of LBW. In rapidly developing countries, such as China, the highest levels of air pollution may be of concern for both preterm birth and LBW.
