# Erratum: “Outdoor Air Pollution, Preterm Birth, and Low Birth Weight: Analysis of the World Health Organization Global Survey on Maternal and Perinatal Health”

**DOI:** 10.1289/ehp.122-A151

**Published:** 2014-06-01

In Figure 1 of the article “Outdoor Air Pollution, Preterm Birth, and Low Birth Weight: Analysis of the World Health Organization Global Survey on Maternal and Perinatal Health” by Fleischer et al. [Environ Health Perspect 122:425–430 (2014); http://dx.doi.org/10.1289/ehp.1306837], clinics in Algeria were omitted from the map of Africa (center). In addition, clinics in other countries that were excluded from the analysis as a result of data quality issues should not have been included in the map. The corrected figure appears below.

The authors regret the error.

